# Synthesis of Co_3_O_4_ Cotton-Like Nanostructures for Cholesterol Biosensor

**DOI:** 10.3390/ma8010149

**Published:** 2014-12-31

**Authors:** Sami Elhag, Zafar Hussain Ibupoto, Omer Nour, Magnus Willander

**Affiliations:** 1 Department of Science and Technology, Campus Norrköping, Linköping University, SE-60174 Norrköping, Sweden; E-Mails: omer.nour@liu.se (O.N.); magnus.willander@liu.se (M.W.); 2 Dr. M.A. Kazi Institute of Chemistry, University of Sindh Jamshoro, Sindh 76080, Pakistan; E-Mail: Zaffar_Ibupoto@yahoo.com

**Keywords:** C_3_O_4_ nanostructures, surfactant, hydrothermal synthesis, potentiometric processes, cholesterol biosensor

## Abstract

The use of templates to assist and possess a control over the synthesis of nanomaterials has been an attractive option to achieve this goal. Here we have used sodium dodecyl sulfate (SDS) to act as a template for the low temperature synthesis of cobalt oxide (Co_3_O_4_) nanostructures. The use of SDS has led to tune the morphology, and the product was in the form of “cotton-like” nanostructures instead of connected nanowires. Moreover, the variation of the amount of the SDS used was found to affect the charge transfer process in the Co_3_O_4_. Using Co_3_O_4_ synthesized using the SDS for sensing of cholesterol was investigated. The use of the Co_3_O_4_ synthesized using the SDS was found to yield an improved cholesterol biosensor compared to Co_3_O_4_ synthesized without the SDS. The improvement of the cholesterol sensing properties upon using the SDS as a template was manifested in increasing the sensitivity and the dynamic range of detection. The results achieved in this study indicate the potential of using template assisted synthesis of nanomaterials in improving some properties, e.g., cholesterol sensing.

## 1. Introduction

Cobalt oxide is an important multifunctional material and it has three well-known polymorphs [1]: the cobaltous oxide (CoO), the cobaltic oxide (Co_2_O_3_) and the cobaltosic oxide (Co_3_O_4_). Compared to the other two polymorphs, Co_3_O_4_ has attracted great interest owing to its potential application in energy storage [2] heterogeneous catalysts [3], and electrochromic devices [4]. Intrinsic Co_3_O_4_ is a p-type semiconductor and has both direct and indirect band gaps of 2.10 eV and 1.60 eV, respectively [2,5]. It crystallizes in a spinel structure with the Co^3+^ ions occupying the octahedral sites, and a Co^2+^ ions occupying tetrahedral sites with the oxygen ions forming a close-packed face centered cubic lattice [6]. This arrangement of ions leads to the fact that the Co_3_O_4_ has explicit surface features with different polar terminations [7]. In addition, Co_3_O_4_ has a paramount electro-catalytic activity [8,9,10,11] and relatively high isoelectric point (IEP) value ~8 [12]. These properties make Co_3_O_4_ constitute a suitable matrix to immobilize some enzymes with relatively low IEP through electrostatic attraction, e.g., cholesterol oxidase with IEP value of ~4.6.

In Co_3_O_4_ nanostructures the sensing mechanism is mainly due to redox-activity at the surface, which can eventually be distinguished as a physisorption or chemisorption activities. Usually point defects are playing a curial role in this adsorption [13]. In addition, the size also demonstrates catalytic activity dependency, *i.e.*, increased sensitivity with decreasing diameter [14]. Therefore, in order to enhance the sensitivity and selectivity of sensors, controlling the morphology provides an important scenario. Furthermore, the approach of incorporating a small amount of impurities to enhance catalytic activity is well established [15]. For example in some metal oxides semiconductors palladium or platinum were predominantly the dopants used for enhancing gas sensing [16]. The same principle can be applied to biosensors [17]. On the other hand, abnormal levels of cholesterol are related to be the cause of atherosclerosis and heart diseases [18,19]. Since the early 1940s, many attempts have been made for the development of an efficient cholesterol biosensor [20].

The objective of this work is to tailor the morphology and modify the charge transfer properties of Co_3_O_4_ nanostructures synthesized by the hydrothermal low temperature method using sodium dodecyl sulfate (SDS) as a template. The synthesized material was then used to fabricate a cholesterol sensor with enhanced properties compared to sensors fabricated using Co_3_O_4_ synthesized without the use of the SDS. This surfactant is known to possess unique ability to self-assemble into aggregates, such as micelles, which serve as templates during the synthesis process [21]. Nowadays, SDS has found use in the development of current technology owing to it’s an amphipathic structure that has a significant role on modifying the properties of surfaces [22]. It has been exploited as a biodegradable [23], and an inhibitor for copper corrosion [24].

## 2. Results and Discussion

### 2.1. *Morphological and Structural Characteristics*

**Figure 1:**
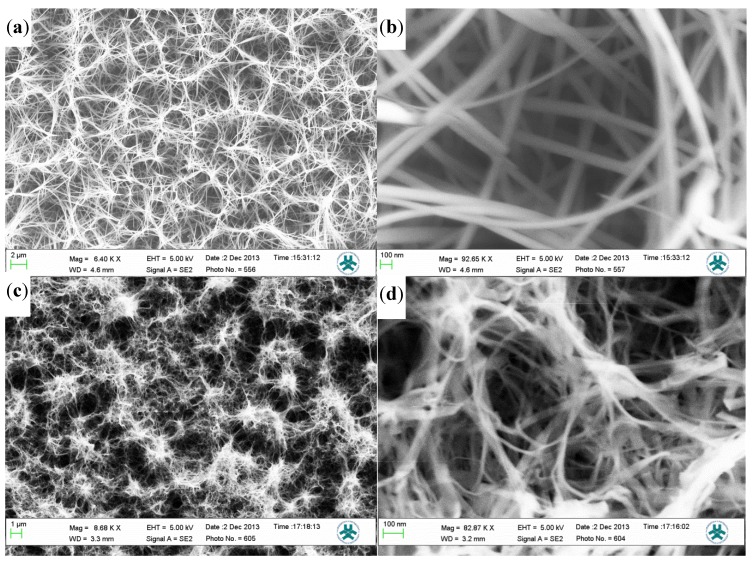
SEM images of (**a**,**b**) Co_3_O_4_ wire-like nanostructure synthesized without the SDS; In (**c**,**d**) Co_3_O_4_ cotton like-nanostructures synthesized with 10 mM SDS is shown.

Scanning electron microscope (SEM) images in Figure 1a,b show the morphology of the Co_3_O_4_ synthesized without the use of the SDS. While in Figure 1c,d a sample synthesized by adding 10 mM SDS is shown. As can be clearly seen, without the use of the SDS, the morphology is rather similar but with relatively smaller dimensions. The SEM images of the Co_3_O_4_ synthesized using 10 mM SDS resulted in relatively very small diameter (~less than 20 nm) and were relatively longer compared to those synthesized without the SDS. This is in consistence with the results published in [11,25,26]. In these articles it was reported that nanostructures morphology is governed by the change of the surfactant. Moreover, Narayan *et al.* [11] used the same approach as we report here; however, their investigation is based on nano-clusters of cobalt oxide. The effect of the SDS on the synthesis mechanism is not completely understood. However, we propose that the function of the surfactant molecules have a direct influence on the thermodynamics of the system. Since the whole system is subjected to 90 °C it will have an influence on the micelles aggregation of the SDS [21]. Therefore, the SDS acts as a synthesis template and would initiate the synthesis of the nanostructures.

**Figure 2:**
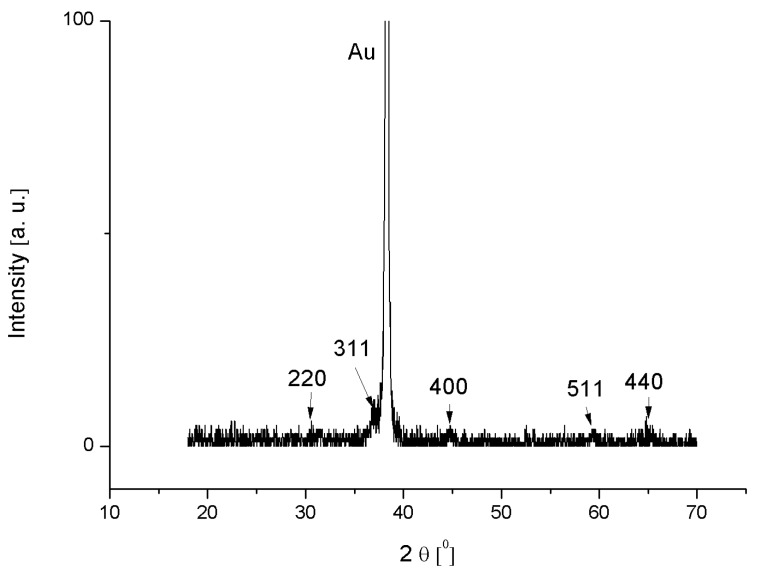
The XRD spectrum of C_3_O_4_ nanostructures on an Au-coated glass substrate.

X-ray powder diffraction (XRD) pattern of the Co_3_O_4_ nanostructures is shown in Figure 2. All the peaks have been assigned to Miller indices that are consistent with the standard spectrum (JCPDS 42-1467) of cubic crystalline Co_3_O_4_. The intensities of the Co_3_O_4_ nanostructures peaks are less when compared to the high intense peak at ~38°, which is related to Au. Thus, it is clear that the SDS has no effect on the crystal orientation.

**Figure 3:**
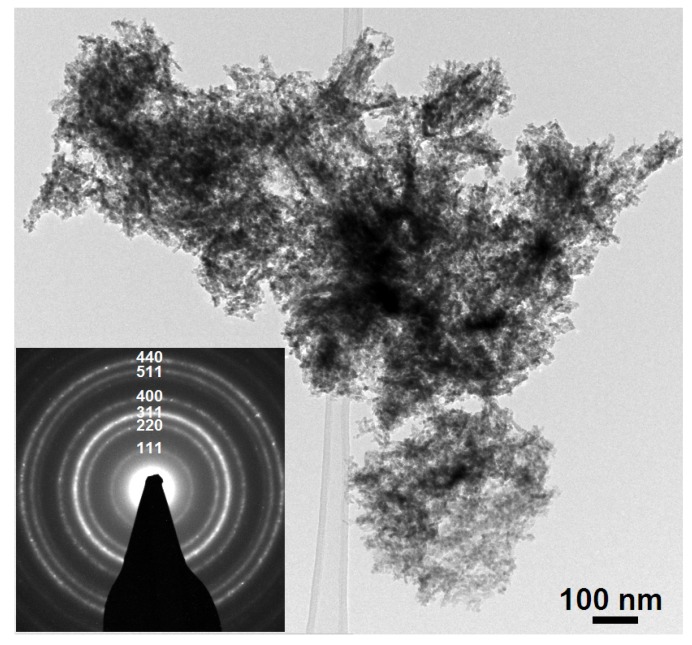

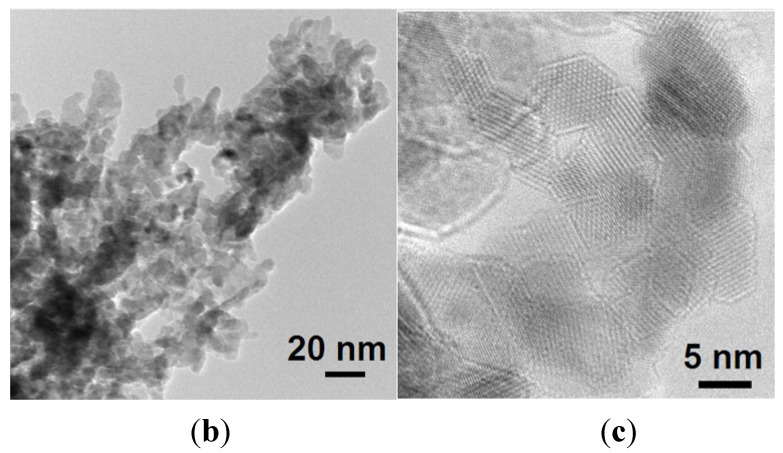
(**a**) a low magnification bright field TEM images of Co_3_O_4_ nanostructures with an insert showing a selected area electron diffraction (SAED) pattern; (**b**) HRTEM image showing Co_3_O_4_ nanoparticles of; (**c**) HRTEM image of Co_3_O_4_ nanoparticles.

Moreover, in order to confirm the XRD results, crystallographic analysis was performed using high-resolution transmission electron microscopy (HRTEM). Figure 3 shows HRTEM images of Co_3_O_4_ prepared with 3 mM SDS. From the selected area electron diffraction (SAED) inserted into Figure 3a it can clearly be seen that the Co_3_O_4_ nanostructure is cubic. As we can see from Figure 3a,b the Co_3_O_4_ nanostructures consist of nanowires with rather small diameters. The HRTEM image shown in Figure 3c reveals that the nanoparticles are crystalline Co_3_O_4_ nano-grains, a fact that is in good agreement with other published results [27].

**Figure 4:**
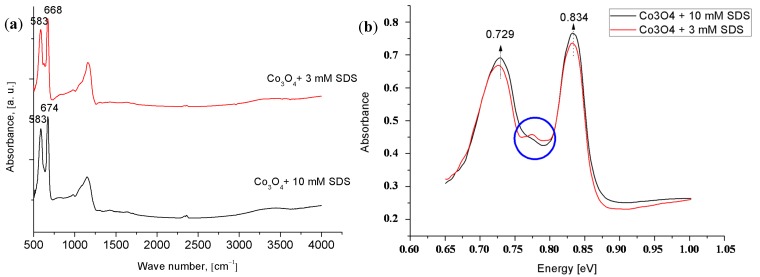
IRRAS spectra ((**a**) wave number; (**b**) energy) of the Co_3_O_4_ nanostructures synthesized with different SDS concentrations 3 mM (red line) and (black line) 10 mM.

Infrared reflection-absorption spectroscopy (IRRAS) was used to investigate more closely the nanostructures presence and further confirm that the adsorbed surfactant molecules are removed from the Co_3_O_4_ nanostructures surfaces. The IRRAS result is shown in Figure 4. The absorption bands at 583, 583, and at 668, 674 cm^−1^ were obtained from Co_3_O_4_ nanostructures synthesized using SDS amount of 3 and 10 mM, respectively. These peaks were associated to the stretching vibrations of the Co^3+^ and Co^2+^-oxygen bond, respectively. These two bands were also observed in other published research on Co_3_O_4_ nanostructures [28]. There is no direct evidence of the presence of the SDS molecules on the surfaces of the synthesized Co_3_O_4_ nanostructures. From the insert spectrum in Figure 4, we noticed a slight variation between the bands “in the circle”. This might be attributed to the fact that the metal cation bonds are affected by the anionic SDS [29]. This effect is called intervalence charge transfer and it yields a new charge transfer processes [12,30].

### 2.2. Cholesterol Biosensor Performance

**Figure 5:**
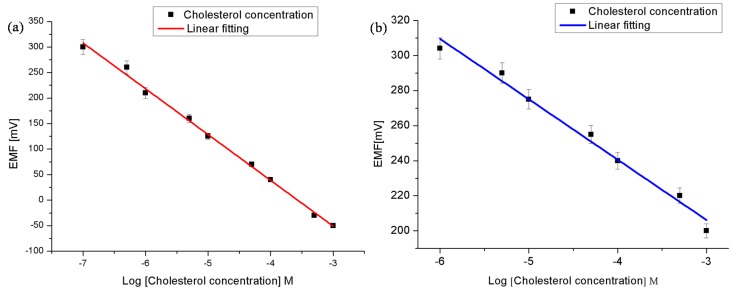
(**a**) Shows a calibration curve of the cholesterols biosensor by using SDS as the synthesis template. The linear calibration equation is given by *y* = −89.76*x* − 320.2, and (**b**) Shows a calibration curve of the cholesterols biosensor without the addition of the SDS, and the linear calibration equation is given by *y* = −34.4*x* + 102.8.

**Figure 6:**
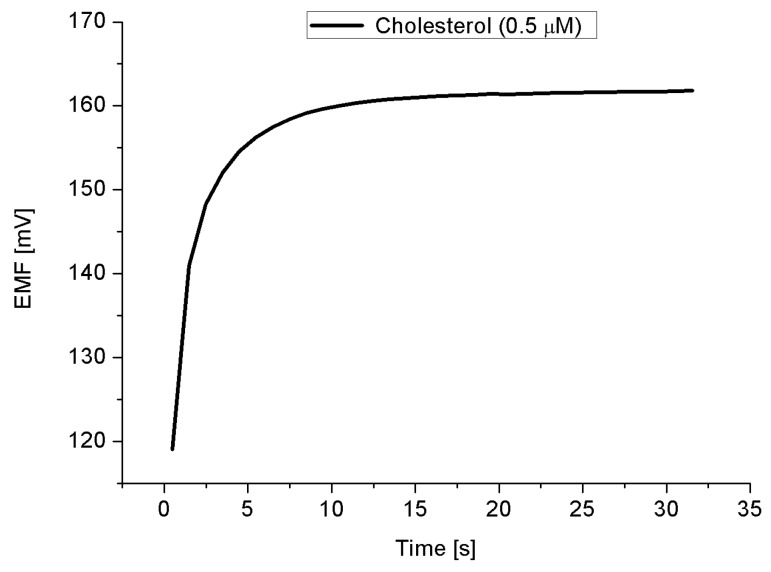
The time response of cholesterol biosensor in concentrations of 5 × 10^−6^ M of Cholesterol.

The obtained results revealed that the proposed cholesterol oxidase/Co_3_O_4_ cotton-like nanostructures/Au electrode possesses a linearity behavior as clearly shown in Figure 5a, with a sensitivity around −90 mV/decade in a concentration ranged from 1 × 10^−7^ and up to 1 × 10^−3^ M. The electromotive force is altered when the composition of the phosphate buffer solution is modified by the cholesterol concentration. We tested Co_3_O_4_ nanostructures electrode from material synthesized without the SDS and we found that the linear range was reduced to be from 1 × 10^−6^ to 1 × 10^−3^ M and the sensitivity was about −34 mV/decade (Figure 5b). The value of this sensitivity constitutes a significant reduction compared to samples synthesized using the SDS, *i.e.* reduction of ~−56 mV/decade. A response time of less than 10 s was observed as depicted in Figure 6. The calculated lower limit of detection was found to be 0.036 × 10^−7^ M. During the experiments the cholesterol biosensor based on the Co_3_O_4_ nanostructures have shown a Nernst response given by:

E = E_0_ − 0.05916V/n log [Reduced]/[Oxidized] (1)

where E is the output potential of working electrode (such as the cholesterol oxidase immobilized Co_3_O_4_ nanostructures); E_0_: is the constant output potential of the reference electrode (Ag/AgCl); and n is the number of electrons involved in the oxidation of the cholesterol. Depending on the type of the electrode being n- or p-type semiconductor the Nernst curve would have a positive or negative value, respectively. Since we have a p-type electrode, the response signal is expected to have high value for a low concentration of cholesterol, and this response will decrease as the concentration is increased. Indeed this is consistent with our observation. The high sensitivity of the presented cholesterol sensor based on the Co_3_O_4_ nanostructures could be attributed to their large surface area to volume ratio, which carried high degree of the cholesterol oxidase molecules and exposing a large surface for the oxidation of the cholesterol molecules on the surface of the enzyme immobilized Co_3_O_4_ nanostructures [31]. The superior sensitivity of the SDS synthesized nanostructures enhancement compared to those synthesized without the SDS may be due to the impurities and electrostatic formation into the structure upon an addition of the anionic surfactant [32,33,34]. Also the SDS could maximize the catalytic activities as have been reported with Narayan *et al.* [11]. Furthermore, in our approach, we believe that, the dehydration of the cobalt hydroxide phase gives rise to the creation of point defects, which would play a very important role in the determination of the catalytic activity of the Co_3_O_4_. This explanation is also consistent with the IRRAS results (the inset Figure 5).

**Figure 7:**
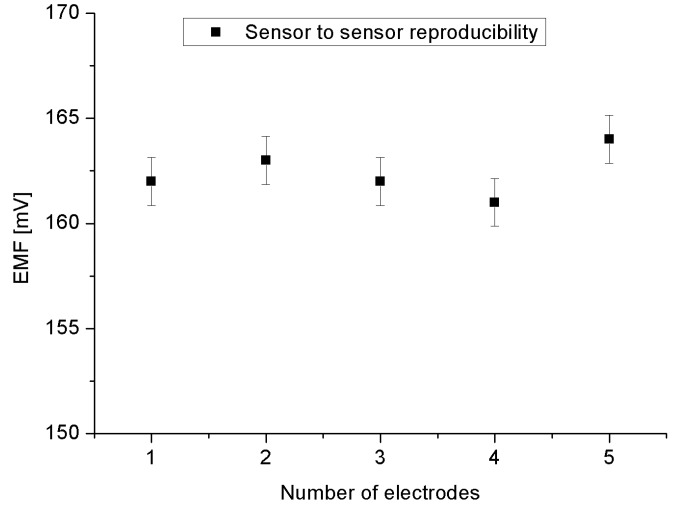
The reproducibility for five working electrodes in a 5 × 10^−6^ M of cholesterol.

An electrochemical sensor performance is usually evaluated by different parameters [35]. The reproducibility is a crucial property of the chemical sensor for knowing its robustness and repeatability. In order to study the reproducibility for the proposed sensor, we independently constructed five sensor electrodes synthesized using the same synthesis and functionalized conditions and procedure, respectively. We have checked the responses of all five prepared sensors in a 5 × 10^−6^ M cholesterol concentration. The result is shown in Figure 7 with a relative standard deviation of less than 5%. This robustness can be understood via the fact that the Co_3_O_4_ cotton-like nanostructures have a high IEP value that have led the absence of difficulty for the direct electron transfer between the enzyme’s active sites and the electrode. Moreover, Co_3_O_4_ cotton-like nanostructures/Au bilayer can even acquire improved electrochemical activity [36].

**Figure 8:**
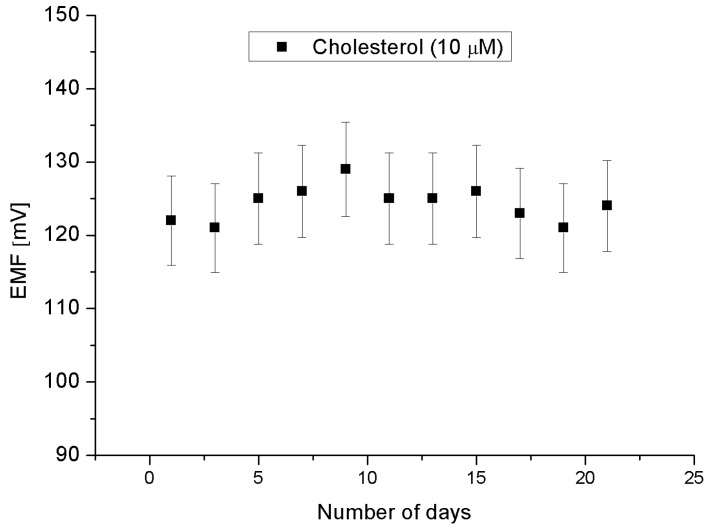
Repeated experiments for eleven days during three weeks in a 10 × 10^−6^ M cholesterol solution using same electrode.

**Figure 9:**
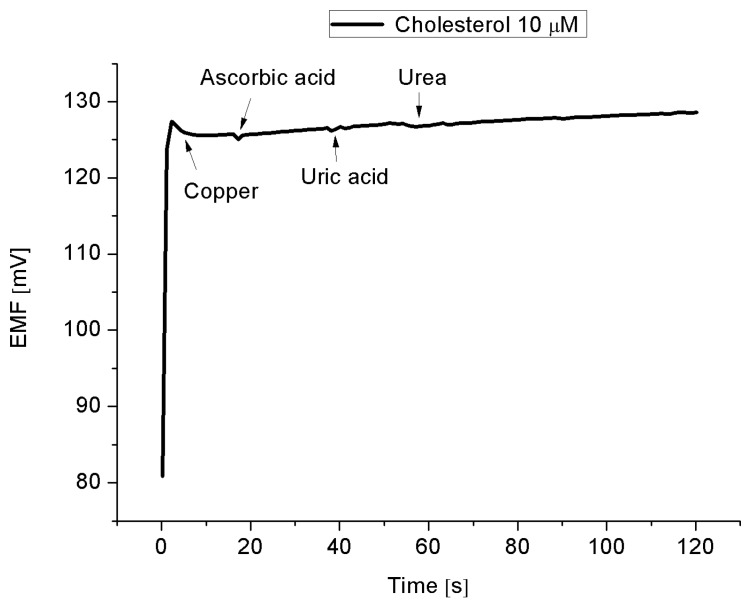
The EMF as a function of time with adding 0.5 mM copper, ascorbic acid, uric acid, and urea, respectively, to a 10 × 10^−6^ M cholesterol solution.

The stability and lifetime, also important for evaluating sensors, have been confirmed by one sensor over three weeks period and is plotted in Figure 8. These results are for performing measurements every two days. During these eleven independent experiments, it was observed that the proposed sensor exhibited more or less a stable signal, *i.e.*, maintaining the sensitivity and response time with a relative standard deviation of less than 5%. All the measurements have been performed at a pH of around 7.3. Therefore, it can be inferred that the enzymatic sensor can be used in these periods of time without any discrepancy in the detection or sensitivity. The selectivity is also a basic parameter for the description of a selective response for cholesterol molecule in the presence of other common interferents. Figure 9 shows the results of experiments of addition of 0.5 mM copper, ascorbic acid, uric acid, and urea, respectively to 0.01 mM cholesterol solution. The results indicate that the interference of the added elements do not significantly modify the signal intensity, indicating the cholesterol oxidase efficient selectivity.

## 3. Experimental Section

### 3.1. Chemicals

Cobalt chloride hexahydrate, urea, cholesterol oxidase, cholesterol, glucose, uric acid, ascorbic acid, copper nitrate, potassium dihydrogen phosphate, disodium hydrogen phosphate, potassium chloride, and sodium chloride were purchased from Sigma Aldrich Sweden. All chemicals were of analytical grade reagents and were used without further purification.

### 3.2. Synthesis of Co_3_O_4_ Nanostructures

The hydrothermal method was used for the synthesis of cobalt oxide nanostructures on gold (Au) coated glass substrate using SDS surfactant as a synthesis template. Some glass substrates were sonicated in ultrasonic bath for about 10 min in acetone-deionized water, and in isopropanol, respectively. Then these substrates were dried by an air gun. The substrates were then affixed into a vacuum chamber of an evaporator instrument Satis CR 725. After this an adhesive layer of 20 nm of titanium was evaporated on the substrates and then a 100 nm thickness layer of gold thin film was evaporated. After that the substrate was affixed upside down in the Teflon sample holder and dipped in 100 mL deionized water solution, containing urea and cobalt chloride in equimolar concentration (0.1 M) and 10 mM of the SDS for 5 h in a preheated electric oven at 90 °C. The amount of the added SDS was also varied in other samples in order to reveal the effect of the SDS. The substrates were seeded with cobalt acetate anhydrous layer via spin coating technique at 1000 rpm. This process was repeated three times and then the samples were annealed at a temperature of 120 °C for 5 min. After completion of the synthesis duration, the samples were washed with deionized water and dried at room temperature. The resulting cobalt hydroxide nanostructures were annealed at 450 °C for 3 h to convert the hydroxide phase of cobalt to the crystalline oxide phase [1]. This annealing step also ensures the removal of the surfactant from the surface of the nanostructures. The possible reactions involved in the synthesis of cobalt oxide nanostructures with the use of SDS as a template can be represented by the following equations:

CoCl_2_ → Co^2+^ + 2Cl^−^ (2)

(H_2_N)_2_ CO + H_2_O → 2NH_3_ + CO_2_ (3)

NH_3_ + H_2_O → NH^+^_4_ + OH^−^ (4)

Co^2+^ + 2OH^−^ + SDS → Co(OH)_2_ (5)

### 3.3. Instrumentations

The morphology and structural properties of the Co_3_O_4_ nanostructures were studied by using LEO 1550 Gemini field emission scanning electron microscope (SEM, Zeiss, Germany) running at 5 kV. The crystal quality of the cobalt oxide nanostructures was studied by X-ray powder diffraction (XRD, Almelo, The Netherland) using a Phillips PW 1729 powder diffractometer equipped with CuKα radiation (λ = 1.5418 Å) using a generator voltage of 40 kV and a current of 40 mA. High-resolution transmission electron microscopy (HRTEM, Hillsboro, OR, USA) experiment was carried out by using a FEI Tecnai 2G UT instrument operated at 200 kV with point resolution of 1.95 Å. Fourier transforms infrared analysis was performed using Bruker Equinox 55. The Infrared Reflection-Absorption Spectroscopy (IRRAS, Billerica, MA, USA) spectrum was the sampling technique and was measured by using grazing angle reflection ~82° with a clean Au (100 nm) sample as a reference.

### 3.4. Immobilization of Co_3_O_4_ Nanostructures with Cholesterol Oxidase and Electrochemical Measurements

**Figure 10:**
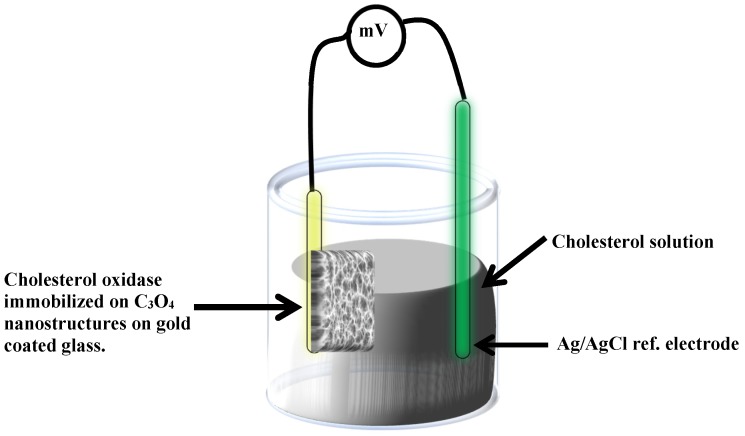
Illustration for the present cholesterol biosensor adopting the potentiometric measurement approach using cholesterol oxidase immobilized cobalt oxide nanostructures.

The electrochemical measurements (see Figure 10 depicts the potentiometric procedure for enzymatic cholesterol biosensors) have been done by immobilizing cholesterol oxidase on the Co_3_O_4_ nanostructures based electrodes and were measured against a silver-silver chloride as a reference electrode at room temperature using a Keithley 2400. A cholesterol oxidase solution was prepared in a 10 mM phosphate buffer of pH 7.3. Cholesterol oxidase was physically adsorbed on the Co_3_O_4_ nanostructures surfaces through electrostatic attraction using drop casting and the electrodes were dried overnight at room temperature inside a fume hood. After that, all the functionalized sensor electrodes were kept at 4 °C when not in use. A stock solution of 50 mM of cholesterol was prepared in few drops of isopropanol and finally mixed with10 mM phosphate buffer of pH 7.3. The different concentrations of cholesterol for testing the sensor were obtained by dilution.

## 4. Conclusions

In summary, we have demonstrated that surfactant assisted synthesized Co_3_O_4_ possesses structural properties that can lead to enhance its sensing properties. The SDS has a dual effect on the synthesis of Co_3_O_4_ nanostructures. Cotton-like Co_3_O_4_ nanostructures have been achieved with a relatively smaller diameter compared to samples synthesized without the SDS. In addition, for the SDS synthesized sample a slight variation in the Co^2+^-Co^3+^ charge-transfer processes has been noticed. An achieved sensitivity of −90 mV/decade, fast response time of ~10 s, and lower limit of detection of 1.0 × 10^−7^, were measured and found to be superior to those obtained from samples synthesized without the SDS. Further, the SDS prepared sensors were found to be reproducible, repeatable, and stable. Further, it was found that hey act as a selective enzymatic cholesterol biosensor. The presented results indicate that the use of surfactant-assisted synthesis of nanostructures can provide a route for further increase of the functionality of chemical sensors.
